# Acute Effect of Simultaneous Exercise and Cognitive Tasks on Cognitive Functions in Elderly Individuals with Mild Cognitive Impairment

**DOI:** 10.3390/diseases12070148

**Published:** 2024-07-10

**Authors:** Ines Ben Ayed, Achraf Ammar, Mohamed Ali Boujelbane, Atef Salem, Salma Naija, Sana Ben Amor, Khaled Trabelsi, Haitham Jahrami, Hamdi Chtourou, Yassine Trabelsi, Farid El Massioui

**Affiliations:** 1 Research Laboratory, Exercise Physiology and Physiopathology: From Integrated to Molecular “Biology, Medicine and Health”, LR19ES09, Faculty of Medicine of Sousse, Sousse University, Sousse 4000, Tunisia; ines.benayed@isseps.usf.tn (I.B.A.); trabelsiyassine@yahoo.fr (Y.T.); 2 Laboratory of Human and Artificial Cognition (EA 4004), Psychology UFR, University of Vincennes/Saint-Denis, 93200 Saint-Denis, France; 3 Research Laboratory, Education, Motricity, Sport and Health (EM2S), LR15JS01, High Institute of Sport and Physical Education of Sfax, University of Sfax, Sfax 3000, Tunisia; trabelsikhaled@gmail.com; 4 Department of Training and Movement Science, Institute of Sport Science, Johannes-Gutenberg-University Mainz, 55122 Mainz, Germany; mboujelb@uni-mainz.de (M.A.B.);; 5 Research Laboratory, Molecular Bases of Human Pathology, LR19ES13, Faculty of Medicine of Sfax, University of Sfax, Sfax 3000, Tunisia; 6 High Institute of Sport and Physical Education of Sfax, University of Sfax, Sfax 3000, Tunisia; h_chtourou@yahoo.fr; 7 Neurology Department, University Hospital Sahloul Sousse, Sousse 4052, Tunisia; naijasalma@gmail.com (S.N.); kaffelsana@yahoo.fr (S.B.A.); 8 College of Medicine and Medical Science, Arabian Gulf University, Manama 293, Bahrain; hjahrami@health.gov.bh; 9 Research Unit, Physical Activity, Sport, and Health, UR18JS01, National Observatory of Sport, Tunis 1003, Tunisia

**Keywords:** physical exercise, simultaneous combined exercise, adults with MCI, attention, memory and problem solving

## Abstract

The increasing prevalence of age-related cognitive decline, alongside the aging global population, underscores the urgent need for innovative and effective preventative strategies. While the advantages of combining physical and cognitive exercises have been recognized as a promising approach to address these socioeconomic challenges, the acute effects of such interventions on cognitive functions remain understudied. This study aimed to investigate whether simultaneous physical and cognitive exercise has a greater beneficial impact on the cognitive functions of older adults with mild cognitive impairment (MCI) than physical exercise alone or reading activities. A total of 44 MCI patients (75% females aged between 65 and 75 years) were randomly assigned to one of three groups: aerobic exercise alone (EG group, *n* = 15), aerobic combined with cognitive exercises (CEG group, *n* = 15), or a reading task for controls (CG group, *n* = 14). Attention, memory, and problem solving were assessed before and after the acute intervention using the Tower of Hanoi, Digit Span, and Stroop tasks, respectively. Statistical analysis revealed that both of the experimental interventions appeared to enhance cognitive function scores (*p* < 0.05), except for the number of moves in the Tower of Hanoi task, where no improvement was noted. In contrast, no significant differences in any cognitive performance measures were observed following the reading session. Notably, the CEG group exhibited a more pronounced positive impact, especially on working memory. This advantage was specifically evident in the digit span tasks, where significantly greater percentage gains were found in the CEG than in the CG (*p* = 0.02), while no significant difference existed between the EG and CG. Simultaneous combined exercise has proven to be a more effective method than aerobic physical exercise alone for improving cognitive function. The results of this study are recommended for inclusion in clinical practice guidelines to maintain the mental health of older adults, as simultaneous exercise seems to offer a time-efficient strategy to enhance cognitive performance in adults with MCI.

## 1. Introduction

Given the increasing aging population in many low- and middle-income countries, the prevalence of dementia is expected to double within the next 20 years [1]. Approximately 1 in 10 older adults aged 70–74 years have mild cognitive impairment (MCI), with prevalence rates increasing with age [2]. MCI is defined as the stage between the expected cognitive decline associated with aging—which includes preserved daily activities—and the more severe cognitive decline characteristic of dementia [3]. Middle-aged and elderly individuals with MCI typically retain functional capabilities that align with expectations for their age and educational level. However, they experience cognitive declines that exceed those typical for their demographic characteristics [2]. Conversion rates from MCI to dementia vary across studies; however, individuals diagnosed with MCI are more likely to progress to dementia than are those with normal cognitive functions [2]. A recent study revealed that approximately 14.9% of individuals aged 65 years and older with MCI developed dementia within a two-year period [2]. Experts in this field argue that identifying MCI provides a crucial opportunity to enhance protective factors and reduce risk factors before functional abilities are impaired [4].

Despite this, the existing body of evidence offers limited high-quality support for the effectiveness of pharmacological interventions in patients with MCI [5]. Although certain medications are approved for treating dementia, their efficacy in improving cognitive functions in MCI patients has not been conclusively proven [5]. Consequently, there is increasing interest in exploring nonpharmacological therapies to enhance cognitive function in MCI patients [5]. Modifiable risk factors, such as poor cardiovascular health, social isolation, depression, and particularly physical or cognitive inactivity, have been estimated to contribute to approximately 40% of the risk for developing dementia throughout the lives of healthy older adults [5]. These factors are recognized as important targets for nonmedical approaches aimed at preventing, reducing, and even delaying cognitive deficits. For many years, a wealth of studies has explored the acute effect of aerobic exercise on cognition. 

This effect has been extensively investigated in both healthy young adults [6,7,8] and older adults [9,10,11,12]. The main finding is that a single session of aerobic exercise has a beneficial effect on executive functions [9,10], visual perception [10], attention [11], and inhibitory control [12]. To the best of our knowledge, the study by Ji et al. [12] is the first to use four experimental conditions involving a sedentary reading control group, physical exercise, cognitive exercise, and combined physical and cognitive exercise in healthy older adults. Surprisingly, these authors did not demonstrate a greater effect of acute simultaneous combined exercise on cognitive functions. However, they successfully improved executive performance following physical exercise alone and combined but not after cognitive exercise alone or during the reading condition. Interestingly, they also reported that exercise led to greater oxygenation of the prefrontal cortex after combined exercise than after other experimental sessions. These results contrast with those recently reported by Pellegrini-Laplagne et al. [13], who reported an improvement in executive performance after each exercise condition, with a particularly greater effect on flexibility following simultaneous combined exercise. These authors also observed a decrease in cerebral oxygenation in both hemispheres after each intervention across all cognitive performance assessments in healthy older adults [13].

In addition to studies examining the effects of acute exercise in healthy older adults, significant attention has been focused on its specific impacts on cognitive function in individuals with mental disorders [14,15]. Mild cognitive impairment (MCI) and Alzheimer’s disease (AD), a specific form of dementia, are the most prevalent mental disorders known to impair cognitive functions—including executive functions, working memory, planning, and inhibitory control—and negatively impact the quality of life and autonomy [16]. Therefore, identifying effective interventions to enhance cognitive function in patients with MCI and thereby reduce the progression to dementia remains a critical scientific endeavor in the field of geriatric health [5]. Although medical treatments for MCI and AD show promise, they often have undesirable side effects [16]. Nonpharmacological interventions, such as exercise, play a crucial role in managing MCI and AD because of the benefits that acute exercise can have on cognitive functions, particularly executive function [16]. 

Recent findings by Chang et al. [17] suggest that acute exercise could be advocated as a lifestyle intervention to enhance cognitive functions in populations at risk for Alzheimer’s disease. Similarly, the study by Ben Ayed et al. [15] demonstrated the positive impact of acute moderate aerobic exercise on cognitive functions—attention, memory, and executive function—in patients with moderate AD, with the most significant effects observed in simultaneous combined exercise compared to physical exercise alone or a sedentary reading control group. Concurrently, Lo et al. [16] showed that acute moderate aerobic exercise improved inhibitory control in adults with MCI, whereas high-intensity acute exercise impaired inhibitory control in the same population [16]. Conversely, Devenney et al. [14] reported no positive effect of acute exercise on executive function, thereby attributing the lack of superiority of simultaneous combined exercise on cognitive outcomes in adults with MCI to the high intensity of the exercise used.

Considering the contradictory findings and the equivocal impact of acute physical exercise, recent approaches suggest a more promising effect of combining physical and cognitive acute exercises on cognitive function [18,19]. In this context, the metaanalysis by Zhu et al. [18] highlights the hypothesis that a simultaneous combined strategy could induce greater cognitive improvement than physical or cognitive interventions alone. This hypothesis is supported by a more recent metaanalysis by Gavelin et al. [19], who reported that combined stimulation was more effective in improving cognitive performance than either intervention alone for older adults with and without cognitive impairment. 

Although promising, the available published data on whether a simultaneous combination of physical and mental exercise reliably correlates with improved cognitive performance are limited, especially for individuals who have already developed symptoms of cognitive decline [20]. The aim of the present study was to investigate the acute effect of a single bout of moderate aerobic exercise alone or simultaneously combined with cognitive tasks on cognitive functions in adults with MCI compared to either physical aerobic exercise alone or a sedentary reading intervention. We hypothesized that simultaneous combined physical and cognitive exercise would be beneficial for improving cognitive performance and that the magnitude of improvement would be greater than that with either exercise mode alone.

## 2. Materials and Methods

### 2.1. Study Population

This study involved forty-four adults who were diagnosed with MCI and who volunteered to participate. The diagnosis of MCI was made according to the International Working Group diagnostic criteria (IWG2) [21] at the neurology department of Sahloul Hospital in Sousse, Tunisia. The inclusion criteria were as follows: were between 65 and 75 years old, lived independently in a noninstitutional setting, scored ≥ 26 out of 30 on the Mini Mental State Examination (MMSE) [22], had normal or corrected-to-normal vision and color perception, and were able to move independently on a daily basis without any technical aid. The exclusion criteria included the use of medications that could affect exercise capacity or posture, regular aerobic training in the past six months, cardiovascular or other medical conditions that would prevent physical activity, neurological disorders associated with cognitive decline (including severe cerebrovascular diseases detected by cranial computed tomography or magnetic resonance imaging), and diagnosed severe psychiatric illnesses (e.g., depression).

The experimental protocol was approved by the Research Ethics Committee of the Faculty of Medicine of Sousse, Tunisia (IRB 00008931). All participants provided written informed consent.

### 2.2. Procedure

**Figure 1:**
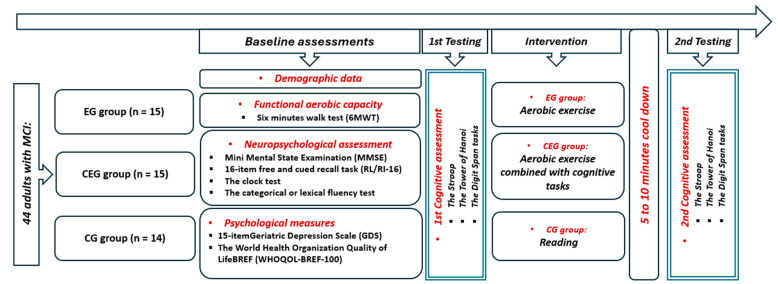
Experimental design.

Participants with MCI were randomly assigned to one of three groups (Figure 1): an exercise group (EG) that participated in a 20 min aerobic physical activity, a combined exercise group (CEG) that simultaneously performed cognitive games while being engaged in the same physical exercise, or a control group (CG) that spent an equivalent amount of time reading. During the aerobic activity, participants in both EG and CEG used a bicycle ergometer (Labyrinth, Paris, France) (http://www.bikelabyrinth.com) (accessed on 1 June 2024) with adjustable seats to accommodate their heights. After a 5 min warm-up, participants in both groups exercised at a moderate intensity for 20 min, thus corresponding to 60% of their maximum heart rate from the 6 min walk test (6MWT). This was followed by a 5 to 10 min cool-down period. For the CEG, while pedaling, participants engaged in cognitive games displayed on a computer screen simultaneously for 20 min of moderate intensity. Cognitive games comprised recreational and simple cognitive tasks that involved various mental processes, such as selective attention, processing speed, scanning, working memory, visual perception, arithmetic, and even temporal perception, all within a single session. Developed by a team of neuropsychologists and medical specialists, these tasks aim to enhance cognitive function in elderly individuals with mild cognitive impairment (MCI). Cognitive assessments were conducted before and after the activities. Tests were administered 5 to 10 min after exercise to ensure that participants’ heart rates had returned to their baseline levels, which were set at 10% above their individual baselines. The cognitive tasks, which assessed attention, memory, and problem-solving skills, were randomly sequenced and counterbalanced to minimize any potential ordering effects. The experiment was overseen by the same individual who provided instructions and was observed without interference during task performance. All sessions were performed at the same time of day (in the morning) to avoid the potential impact of diurnal variation [23,24].

### 2.3. Measurements

#### 2.3.1. Neuropsychological Measures

A comprehensive neuropsychological assessment was conducted for all participants. The MMSE was administered as a paper-based test commonly used in cognitive decline evaluations [22]. The scores on this test range up to 30 points, with a typical cutoff set at 24. Scores between 26 and 30 indicate normal cognitive function, while scores below 25 suggest varying degrees of impairment: mild (20 to 24), moderate (10 to 19), severe (3 to 9), or very severe (below 3) [25].

Episodic verbal memory was evaluated using the 16-item free and cued recall task (RL/RI-16) [26]. This task assesses memory encoding and retrieval processes through free recall, cued recall, and recognition. A normal result is achieved when the total recall equals 16.

Visuoconstructive abilities were assessed using the clock drawing test [26]. Participants were asked to draw a clock showing a specific time (e.g., 10:15). The test is scored out of 7 points, and scores fall into a pathological range if key criteria are not met. These criteria include having all numbers from 1 to 12 present and correctly ordered and having the clock hands appropriately sized and positioned to indicate the correct time.

Verbal fluency was evaluated through categorical or phonemic fluency tasks [27]. Participants were required to generate as many words as possible within 1 min related to a specific category (semantic) or starting with a particular letter (phonemic). Scores are considered pathological if responses fall below 15 for categorical fluency or 10 for phonemic fluency.

#### 2.3.2. Psychological Measures

Mood was assessed using the 15-item Geriatric Depression Scale (GDS) [28]. Scores between 0 and 5 are considered normal. Scores between 5 and 9 indicate a high likelihood of depression, while scores of 10 and above indicate the presence of depression.

Quality of life was assessed using the World Health Organization Quality of Life-BREF (WHOQOL-BREF-100) [29]. This self-report questionnaire consists of 26 items that measure four domains: physical health, psychological health, social relationships, and environmental relationships. According to the World Health Organization, the quality-of-life thresholds for the physical domain are 6–16 (poor), 17–26 (average), and 27–35 (good). For the psychological domain, the thresholds are 6–14 (poor), 15–22 (average), and 23–30 (good). For social relations, the thresholds are 3–7 (poor), 8–11 (average), and 12–15 (good). For the environmental domain, the thresholds are 8–18 (poor), 19–29 (medium), and 30–40 (good). The total score ranges from 26–60 for poor quality of life, 61–95 for average quality of life, and 96–130 for good quality of life [29].

#### 2.3.3. Cognitive Function Measures

In this study, cognitive functions were assessed using the Stroop, Tower of Hanoi, and digit span tasks. These tasks were used as experimental situations rather than tests with pre-established standards. Our goal was to indirectly validate this hypothesis by comparing the performance of participants in the experimental groups to that of participants in the control group.

Selective attention was evaluated using the Stroop test, which is a widely recognized test [30]. This test measures sensitivity to interference and has been adapted in various ways over time (e.g., [31,32,33]). The differences between versions depend on factors such as the number of sheets, number of items, measurement of execution time, and accuracy of responses. Different calculation formulas are used to quantify the performance and describe the effect of interference [34]. In this study, we utilized the Stroop Color–Word Test (Victoria version, French) to measure selective attention. We also added an additional experimental condition to assess automatic reading behavior with a small number of items, thus creating interference conditions [35]. During the test, participants were asked to verbally identify the color of the stimulus presented on a computer screen at a standardized distance. The test consisted of multiple conditions, including identifying the color of rectangles, reading color names written in black, reading color names written in their designated color, and stating the color of a word without reading the word (where the color stated is different from the color written). This task measures concentration and resistance to interference. The stimuli appeared at a speed designed to maintain participant attention, and participants were asked to respond to as many items as possible in each condition. Each condition lasted for 45 s, and we recorded the number of correct items as a measure of performance. Considering that some patients may have physical vulnerability (fatigue) or psychoemotional symptoms, our focus was on the quality of their responses. We quantified the effect of interference by calculating the difference between the number of erroneous responses in the experimental condition and the mean number of erroneous responses in the first three control conditions.

The digit span task was used to evaluate working memory [36,37], specifically the ability to recall a sequence of numbers. In this task, participants are shown digits on a screen at a rate of one digit per second. To pass the forward digit span test, participants need to repeat the sequence in the exact order it was shown. If successful, a new sequence with an additional digit is presented. However, if they fail, they are given a second sequence of the same length. Passing this second sequence results in a longer sequence being shown, while failing it ends the test. The length of the sequences increases from three to nine digits. The backward digit span task follows a similar procedure, but participants must repeat the numbers in reverse order. The longest sequence in this test is eight digits. The final score is determined by the total number of correct sequences completed before experiencing two consecutive failures.

The Tower of Hanoi test was used to assess problem-solving skills [38]. This task involves a wooden stand with three vertical pegs of equal height. Initially, there are three disks of different sizes stacked on the leftmost peg. The goal for participants is to recreate the pyramid of three disks on the rightmost peg, using the middle peg for transferring the disks. There are two rules to follow: a larger disk cannot be placed on top of a smaller disk, and only one disk can be moved at a time.

Before beginning the task, we made sure that participants fully understood the verbal instructions. We recorded both the number of moves made and the time taken to complete the task. The minimum number of moves needed to solve the three-disk Tower of Hanoi is seven.

### 2.4. Statistical Analysis

Descriptive statistics are presented as the mean ± standard deviation (SD). The normality of the data was checked using the Shapiro–Wilk test. To assess the statistical significance of differences between groups for participants’ demographic data, one-way analysis of variance (ANOVA) (when a parametric test was appropriate) or the Kruskal–Wallis test (when a nonparametric test was appropriate) was performed, followed by a post hoc pairwise comparison or Dunn’s test, both with the Bonferroni adjustment. The effect size (ES) statistic (η_p_^2^ and η^2^_H_) was calculated. To assess the statistical significance of differences between groups and measurement times, the F1-LD-F1 model was used. This model provides ANOVA-type statistics for group, time, and the interaction between group and time. When significant main or interaction effects were found, Dunn’s post hoc test with Bonferroni adjustment was performed. To calculate the % of Gain for all parameters, the % of Gain was calculated as follows: % of Gain = (Post − Pre)/Pre)*100. To assess the statistical significance of differences between groups for % of gain in each parameter, the Kruskal–Wallis test was performed. The ES statistic (η^2^_H_) was calculated using the H-statistic. If significant effect of group was found, pairwise comparisons were performed using the Dunn’s test with the Bonferroni adjustment. 

The study utilized Cohen’s d, a standardized effect size analysis, to determine the magnitude of differences between variables. The classification system developed by Hopkins [39] was used to categorize the effect sizes, with values ≤ 0.20 considered trivial, 0.20 < d ≤ 0.60 as small, 0.60 < d ≤ 1.20 as moderate, 1.20 < d ≤ 2.0 as large, 2.0 < d ≤ 4.0 as very large, and d > 4.0 as extremely large. In this study, significance was set at *p* < 0.05 for all analyses, as determined a priori. The statistical analyses were performed using the R programming language [40]. ANOVA and post hoc tests for normally distributed data were conducted with the “afex” [41] and “rstatix” [42] packages, respectively. The Kruskal–Wallis and Dunn’s tests were conducted with the “rstatix” package [42]. The F1-D1-F1 model was performed with the “nparLD” package [43].

## 3. Results

### 3.1. Participant Characteristics

Forty-four adults diagnosed with MCI were randomly assigned to three groups. Baseline assessments confirmed that all participants constituted a homogeneous sample, as the majority of the demographic, physical, and neuropsychological parameters showed no significant differences between the groups (*p* > 0.05) for any of the tested parameters (see Table 1).

### 3.2. Effects on Cognitive Performance: A Comparative Analysis Based on Pre–Post Values

**Figure 2:**
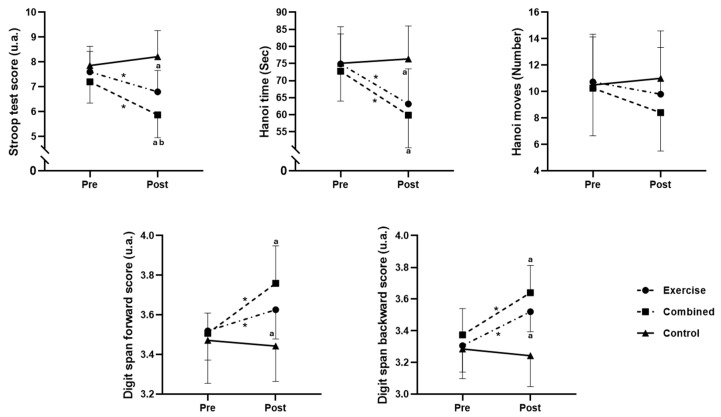
Cognitive performance in the exercise, combined exercise, and control groups at pre- and post-test sessions. *: significant difference between pre- and post-test session values; a: significant difference compared to the control group; and b: significant difference compared to the exercise group.

Pre- and post-test session values for the three tested groups, as well as the differences between groups, are presented in Figure 2.

#### 3.2.1. Stroop Test

The F1-LD-F1 model revealed significant main effects of Group (ATS_mod_ (1.97, 39.56) = 14.12; *p* = 0.0003) and Time (ATS (1, ∞) = 15.31; *p* = 0.0001). Additionally, a significant interaction between group and time was found (ATS (1.95, ∞) = 9.45; *p* < 0.0001). The pairwise comparisons revealed a significant decrease in the Stroop score from pre- to postintervention for the exercise (*p* = 0.0148; d = 0.95) and combined groups (*p* = 0.0017; d = 1.5). Additionally, the Stroop score for the combined group was lower than that for both the exercise (*p* = 0.027; d = 1.05) and control (*p* = 0.0001; d = 1.41) groups postintervention, and the exercise group had a lower Stroop score than the control group (*p* = 0.004; d = 2.38) postintervention (Figure 2).

#### 3.2.2. Hanoi Time

The F1-LD-F1 model showed a significant main effect of Group (ATS_mod_ (1.96, 39.01) = 4.89; *p* = 0.0131) and Time (ATS (1, ∞) = 26.96; *p* < 0.0001). Additionally, a significant interaction between group and time was revealed (ATS (1.95, ∞) = 9.45; *p* = 0.0007). The pairwise comparisons revealed a significant decrease in the Hanoi time from pre- to postintervention for the exercise (*p* = 0.0076; d = 1.11) and combined groups (*p* = 0.0007; d = 1.41). Additionally, the Hanoi time for the combined and exercise groups was lower than that for the control group (*p* = 0.015 and 0.001; d = 1.72 and 1.35, respectively) postintervention (Figure 2).

#### 3.2.3. Hanoi Time

The F1-LD-F1model showed a nonsignificant main effect of Group (ATS_mod_ (1.95, 38.3) = 0.92; *p* = 0.4035) and Time (ATS (1, ∞) = 1.61; *p* = 0.2039). Additionally, a nonsignificant interaction between group and time was revealed (ATS (1.96, ∞) = 1.3; *p* = 0.2721) (Figure 2).

#### 3.2.4. Digit Span Forward

The F1-LD-F1model revealed a significant main effect of Group (ATS_mod_ (1.93, 37.46) = 4.38; *p* = 0.0207) and Time (ATS (1, ∞) = 19.01; *p* < 0.0001). Additionally, a significant interaction between group and time was revealed (ATS (1.93, ∞) = 9.93; *p* < 0.0001). The pairwise comparisons revealed a significant increase in the digit span score from pre- to postintervention for the exercise (*p* = 0.0146; d = 0.72) and combined groups (*p* = 0.0043; d = 1.67). Additionally, the digit span scores for the combined exercise group were greater than those for the control group (*p* = 0.0008 and 0.033; d = 1.73 and 1.08, respectively) postintervention (Figure 2).

#### 3.2.5. Digit Span Backward

The F1-LD-F1 model revealed a significant main effect of Group (ATS_mod_ (1.99, 40.24) = 12.72; *p* < 0.0001) and Time (ATS (1, ∞) = 17.71; *p* < 0.0001). Additionally, a significant interaction between group and time was revealed (ATS (1.89, ∞) = 7.82; *p* = 0.0001). The pairwise comparisons revealed a significant increase in the digit span score from pre- to postintervention for the exercise (*p* = 0.0044; d = 1.44) and combined groups (*p* = 0.0025; d = 1.57). Additionally, the digit span scores for the combined exercise group were greater than those for the control group (*p* = 0.0002 and 0.002; d = 2.16 and 1.57, respectively) postintervention (Figure 2).

### 3.3. Effects on Cognitive Performances: A Comparative Analysis Based on the Percentage of Change from Pre- to Postsession

**Figure 3:**
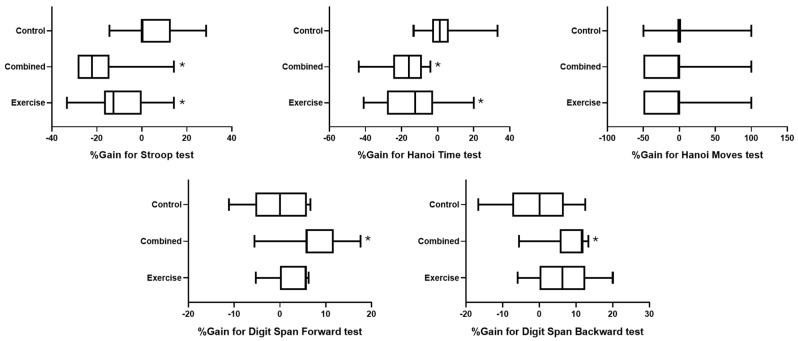
Percentage change in cognitive outcomes for the exercise, combined, and control groups. *: significant difference between pre- and post-test session values.

Figure 3 represents the effect of group on the percentage of change in the different tested cognitive outcomes.

For the Stroop test, the statistical analysis revealed a significant difference between groups (H (2) = 17.5; *p* = 0.0002; ES = 0.378), where the gain was lower in the combined and exercise groups than in the control group (*p* = 0.0002 and 0.018; d = 1.88 and 1.2, respectively) (Figure 3).

Concerning the Hanoi time test, there was a significant difference between groups (H (2) = 16.4; *p* = 0.0003; ES = 0.352), where the gain was lower in the combined and exercise groups than in the control group (*p* = 0.0002 and 0.011; d = 1.83 and 1.33, respectively). However, a nonsignificant difference between groups was found for Hanoi moves (H (2) = 2.6; *p* = 0.272; ES = 0.015) (Figure 3).

For the digit span test, there were significant differences between groups for digit span forward (H (2) = 9.07; *p* = 0.0107; ES = 0.172) and backward (H (2) = 7.46; *p* = 0.024; ES = 0.133), where the scores were greater in the combined group than in the control group for forward (*p* = 0.022; d = 1.43) and backward tests (*p* = 0.027; d = 1.27) (Figure 3).

## 4. Discussion

The present study aimed to investigate the acute effect of simultaneous combined exercise on cognitive functions in adults with MCI compared to either exercise alone or reading activities. Based on the findings of Gavelin et al. [19], we hypothesized that simultaneous physical and cognitive exercise would be more effective in improving cognitive performance, with a superior magnitude of enhancement. In accordance with our hypothesis, we found an improvement in cognitive performance (i.e., attention, working memory, and problem solving) after both exercise conditions but not after the reading activities. This improvement was more pronounced following the combined protocol, particularly in working memory.

For instance, we noted a decrease in interference sensitivity in the Stroop test after exercise among MCI patients. This finding aligns with a study by Ben Ayed et al. [15], who reported that AD patients consistently performed better on the Stroop test after a single 20 min cycling exercise at moderate intensity (60% of the maximum heart rate from the 6MWT) either alone or combined with a cognitive task. However, this result contrasts with the study by Devenney et al. [14], which showed that acute exercise did not improve cognitive performance (sustained attention, executive function, or visuospatial learning and memory) but did increase peripheral sBDNF levels in MCI subjects. The lack of a positive effect of acute exercise on cognitive functions observed by Devenney et al. [14] could be explained by methodological limitations regarding the experimental design, specifically the high intensity of the acute exercise used.

Regarding the Tower of Hanoi task, we observed a significant decrease in execution time following exercise among the experimental groups. This result is consistent with previous research showing that executive function can be enhanced after moderate-intensity exercise (60% of peak oxygen consumption) for a moderate duration (20 min) [44]. Won et al. [45] also highlighted improvements in functional processing and executive function after 30 min of cycling exercise at moderate intensity in older adults. Recently, Pellegrini-Laplagne et al. [13] highlighted the positive effect of simultaneous physical and cognitive exercise (a 30 min session at 60% of the theoretical maximal heart rate) on executive functions in healthy older adults compared to either exercise alone. Additionally, these authors observed a decrease in cerebral oxygenation in both hemispheres following experimental interventions in all cognitive performance tests [13]. This finding is encouraging and suggests that this type of stimulation induces a beneficial effect, thus potentially serving as an efficient cognitive enhancement strategy for elderly individuals.

Concerning the digit span tasks, our findings demonstrated that MCI subjects performing acute moderate aerobic exercises had greater performance than did the control group. This finding is consistent with a previous study [46] in which a 20 min session of moderate cycling exercise improved working memory in older adults. Moreover, in middle-aged adults, a systematic review by Loprinzi et al. [47] showed that acute aerobic exercise had a positive effect on memory. Additionally, a study by Marin Bosch et al. [48] indicated that acute high-intensity exercise significantly improved motor sequence memory, while there was a tendency toward significant enhancement after acute moderate exercise in healthy young males. This improvement was linked to an increase in anandamide (an endocannabinoid known to promote hippocampal plasticity) and was accompanied by local increases in activity within the caudate nucleus and hippocampus. These findings illustrate that acute physical exercise fosters sequence learning, thus underscoring the overall benefit of exercise for memory functions related to the hippocampus [48].

Supporting this notion, Loprinzi et al. [49] specifically examined the impact of acute exercise sessions on short-term and long-term episodic memory. Their analysis of 25 experiments provided insights into the timing of exercise sessions in relation to encoding (before, during, and after), as well as various study parameters such as exercise type, intensity, and duration. Exercise conducted before and after encoding led to improved performance, with larger effects observed when exercise followed encoding compared to before. More recently, a review by Festa et al. [50] showed that proper physical activity and a healthy lifestyle (acute or chronic exercise) improve cognitive functions (memory and executive function) and neurotrophic factors, increase the presence of angiogenic and synaptogenic factors, and reduce inflammatory states and functional connectivity across all age groups.

Interestingly, our results, particularly the larger percentage of gain in working memory compared to the control condition—which has only been observed when engaging in combined exercise—are consistent with previous findings. These studies have highlighted that engaging in combined exercise stimulates consistent exercise toward proper dosing to maximize the cognitive benefits of physical exercise alone [10,15]. A recent metaanalysis by Meng et al. [51] reported that the combination of physical and cognitive interventions demonstrated superiority over single physical exercise or single cognitive intervention in improving executive function (e.g., attention) and memory. Similarly, Gavelin et al. [19] suggested that simultaneous combined interventions are effective in enhancing both physical and cognitive health in healthy older adults, as well as MCI patients, and therefore should be preferred over single-domain interventions.

Additionally, the results of pairwise metaanalyses by Xue et al. [52] indicated that combined interventions were superior to exercise alone in enhancing delayed recall, working memory, and depression in MCI patients. Similarly, Ben Ayed et al. [53] recently reported that physical and simultaneous combined aerobic exercise represent a promising combination for enhancing cognitive performance (e.g., attention, working memory, and problem solving), quality of life, and mood (depression) in MCI patients. These findings are comparable to those of a recently published study by Nath et al. [10], thus indicating that a single bout of interactive physical and cognitive exercise can yield greater cognitive benefits than independent mental or physical exercise.

The synergistic effects of 20 min of pedal-to-play aerobic exercise have been explored in adults with MCI, with participants experiencing greater changes in executive function [54]. Those authors showed that the enhancement of cognitive performance from acute aerobic and combined exercise is likely attributable to greater changes in salivary alpha-amylase levels in the MCI group than in the normative group [54]. Significant increases in biomarkers such as salivary alpha amylase suggest that combined exercise can activate neurobiological mechanisms, which, in other studies, have been linked to facilitated neurogenesis, thus providing an indication of a linking mechanism [50]. Therefore, acute aerobic exercise has been shown to increase the plasma level of BDNF in healthy older adults. as well as in patients with Alzheimer’s disease [55]. It also promotes angiogenesis and the release of neurotrophic factors (e.g., BDNF, VEGF, and IGF-1), which are linked to increased cerebral blood flow and, in turn, promote cerebral plasticity [55]. Additionally, neurotrophic factors are thought to facilitate the clearance of beta amyloid and reduce the hyperphosphorylation of the tau protein, which are key features of AD [55].

### Strengths and Limitations

The present study represents a pioneering effort in assessing the effects of acute bouts of combined physical–cognitive exercise on the cognitive functions of elderly individuals with MCI.

The strength of this study is its innovative approach, thereby demonstrating that a brief, 20 min session of acute aerobic exercise can have a positive impact on the tested parameters and the target population. This duration is shorter than those suggested in earlier protocols [56]. Remarkably, our study observed beneficial effects after only one exercise session. Furthermore, our research is distinguished by its clinical emphasis, thus aiming to maximize cognitive benefits through combined acute interventions, which are considered to be leading nonpharmacological therapies [57]. The group engaging in exercise simultaneously with cognitive tasks exhibited a notably superior effect, especially with respect to working memory.

However, this study presented several limitations. First, while promising, the present results should be interpreted cautiously owing to the small sample size. We recruited only 44 subjects with MCI due to the strict inclusion and exclusion criteria. Second, we did not investigate individual differences from a physiological perspective, and consequently, we could not objectively clarify the mechanisms impacted by acute exercise. In future studies, a larger sample size and the consideration of various neurotrophic factors, such as BDNF or IGF-1, are suggested to improve our understanding.

## 5. Conclusions

In conclusion, compared to reading activities, a single bout of aerobic exercise, either alone or combined with a cognitive task, appears to enhance various cognitive performance outcomes (i.e., attention, working memory, and problem solving), with a more pronounced effect on working memory following the combined protocol. These findings highlight simultaneous combined exercise as a more effective method for improving cognitive functions. The results of the current study may have significant clinical implications, as they enable us to optimize interventions aimed at preserving the cognitive health of older adults. Moreover, they offer a time-efficient strategy to enhance cognitive performance in this population.

## Figures and Tables

**Table 1:** Baseline participant characteristics (*n* = 44).

	Exercise Group (EG, *n* = 15)	Combined Group (ECG, *n* = 15)	Control Group (CG, *n* = 14)	*p* Value	ES
Age (years)	67.93 ± 5.18	67.13 ± 3,04	69.24 ± 4.84	0.444	0 *
Age of the onset of the disease (years)	63.26 ± 4.75	62.00 ± 3.84	64.57 ± 4.44	0.1	0.064 *
Sexe (M/F)	5/10	5/10	2/12		
Neuropsychological measures					
MMSE	26.20 ± 0.56	26.13 ± 0.35	26.07 ± 0.26	0.65	0 *
RL/RI 16	13.13 ± 1.18	13.00 ± 1.25	13.14 ± 1.16	0.937	0.003
Letter fluency	10.53 ± 0.74	10.80 ± 0.77	10.71 ± 0.72	0.561	0 *
Category fluency	13.86 ± 0.99	13.33 ± 0.97	13.07 ± 0.82	0.106	0.061 *
Clock test	6.00 ± 0.53	5.66 ± 0.48	5.57 ± 0.64	0.101	0.063 *
Psychological measures					
GDS 15	5.93 ± 0.45	5.20 ± 0.41	6.21 ± 0.57	0.001	0.451 *
WHOQOL-BREF-100D1	21.00 ± 1.36	21.73 ± 1.83	21.71 ± 1.43	0.354	0.049
WHOQOL-BREF-100D2	20.86 ± 1.30	20.33 ± 1.54	19.35 ± 1.78	0.039	0.147
WHOQOL-BREF-100D3	9.06 ± 1.38	9.67 ± 1.40	9.64 ± 0.74	0.393	0 *
WHOQOL-BREF-100D4	27.00 ± 1.64	26.93 ± 2.37	26.21 ± 2.11	0.535	0.03

MMSE: Mini Mental Status Examination; RL/RI-16: 16-item Free and Cued Recall; GDS: Geriatric Depression Scale; WHOQOL-BREF: World Health Organization Quality of Life-BREF; D1: Physical health; D2: Psychiatry; D3: Social relationships; D4: Environment; *: Effect size calculated by H-statistics value from Kruskal–Wallis test.

## Data Availability

The data are available from the corresponding author upon reasonable request.
